# Shape-controlled movement of Zn/SU-8 micromotors†

**DOI:** 10.1039/d4na00721b

**Published:** 2024-11-25

**Authors:** Tijana Maric, Lasse Højlund Eklund Thamdrup, Anja Boisen

**Affiliations:** a The Danish National Research Foundation and Villum Foundation's Center for Intelligent Drug Delivery and Sensing Using Microcontainers and Nanomechanics (IDUN), Department of Health Technology, Technical University of Denmark Ørsted Plads, 2800 Kgs. Lyngby Denmark tijma@dtu.dk; b Department of Health Technology, Technical University of Denmark 2800 Kgs. Lyngby Denmark

## Abstract

Creating micromotors (MMs) that will have the highest possible velocities has become one of the main focuses in the field of autonomous microdevices research. The importance of velocity stems from various autonomous microdevices applications, ranging from faster drug delivery to the eradication of various bacterial biofilms using only mechanical movement. To investigate how different shapes affect the velocity of Zn/SU-8 micromotors in acid solution, we fabricated micromotors with various geometries (Zn/SU-8/Cylindrical, Zn/SU-8/Rectangular cuboid, Zn/SU-8/Triangular prism, Zn/SU-8/Pentagonal prism and Zn/SU-8/Pentagrammic prism MMs). This is the first comparative study where shape has been isolated as the critical factor influencing micromotor velocity under the same catalytic surface conditions. Our results demonstrate that Zn/SU-8/Rectangular cuboid and Zn/SU-8/Triangular prism MMs exhibit significantly higher average velocities compared to the other studied MMs. The shape-optimized Zn/SU-8 micromotors, characterized by their simple synthesis process and low cost, offer significant potential to enhance efficiency and navigation in both environmental and medical applications through precise movement control.

## Abstract 2

In this study, we investigated how different shapes affect the velocity of Zn/SU-8 micromotors.

## Graphical abstract

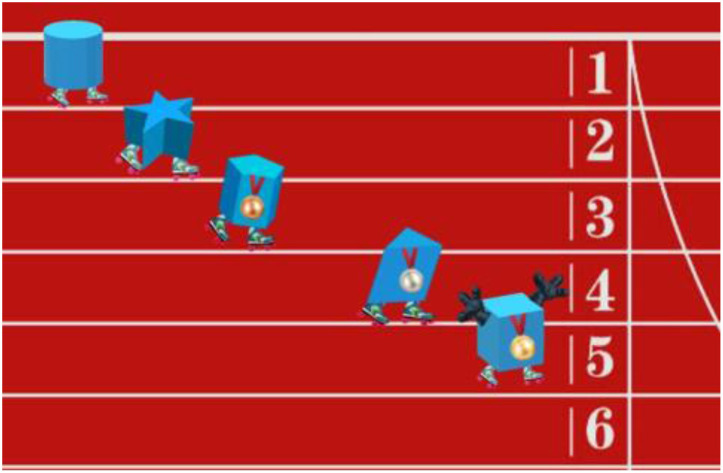

Micromotors (MMs) are usually made of two parts, the first part represents their base, and the second is the catalyst, which is responsible for the reaction of the micromotor with the surrounding environment.^1^ They have mobile properties in the presence of internal fuels (H_2_O_2_, acidic solution, water) or external stimuli (light, ultrasound, magnetic field, electric field).^2–10^ Nowadays, special attention is focused on the fabrication of biocompatible and biodegradable MMs, where they can be used in the field of *e.g.* biomedicine due to their motion abilities.^11,12^ Because of the great variability in size, shape and materials for their production, MMs possess numerous functionalities, which make them extremely attractive not only in biomedical studies but also for environmental remediation.^13,14^ Three common methods employed in the production of MMs are physical vapor deposition, electrochemical deposition, and strain engineering.^15–17^ However, designing MMs in large quantities with different shapes and uniform sizes remains a major challenge.

MMs whose movement can be controlled by geometry are highly desirable and recommended. Their velocity, and therefore their movement, could be affected by two fundamental factors: the environment of the micromotor and the geometry. Changing the geometry (size and shape) is the most efficient way to improve their movement performance.^18^ A previous study demonstrated that convex-shaped micromotors propelled by bubble formation achieve velocities 70% higher than concave micromotors of the same size.^19^ This velocity difference arises from the influence of shape on bubble dynamics, where convex surfaces cause shorter bubble growth and collapse than concave ones. Lee and coworkers experimentally validated the Stokes–Einstein equation, demonstrating that larger micromotors exhibit slower velocities compared to smaller ones.^20^ This size-dependent variation in velocity can be explained by the lower diffusivity associated with larger particles and the greater influence of drag force as the particle size increases.

It has been proven that by improving temperature, reducing the viscosity of the MM medium and raising the concentration of the fuel, the velocity of the MMs can be increased.^21–23^ Bishop and co-workers showed that Pt-microparticles are able to move in hydrogen peroxide solutions by themselves and that their shape can be controlled to optimize their movement.^24^ They presented a mathematical model to explain how the shape of the particles impact the relevant electrocatalytic reaction leading to particle movement by a self-electrophoretic mechanism. Pumera *et al.* studied tubular bubble-propelled nano/micromotors to confirm that the velocity is closely related to bubble ejection, and thus to the size of the created bubbles and expulsion frequency.^25^ Larger tubular MMs have more available catalytic surface area and tend to be more active at high fuel concentration compared to smaller tubular MMs. Similarly, Dong and co-workers explored velocity-controlled behavior of PCL-Pt MMs in H_2_O_2_ fuel.^26^ They demonstrated that the movement depends on the height-width ratio. When the aspect ratio is less than 1, the MMs adopt a vertical configuration and generate oxygen bubbles at their base (bottom), leading to random motion with slower velocities due to less efficient propulsion. In contrast, when the aspect ratio is greater than 1, the MMs have a horizontal configuration, generating bubbles at one end of the structure, which provides unidirectional propulsion, resulting in faster and more efficient movement. It is also possible to control the velocity only by adjusting the power of external stimuli in the case of MMs whose movement is based on the use of external sources (magnetic field, electric field, ultrasound, light).^27,28^ Chen and co-workers demonstrated that the velocity and the direction of magnetic-doped liquid crystal droplets motors could be controlled by an external magnetic field; the velocity could be regulated by increasing the strength of the magnetic field, and the direction could be fixed manually by regulating the orientation of the magnet.^29^ Our group used similar approach to control the velocity of NIR-light-driven mesoporous SiO_2_–Au MMs by adjusting the laser power.^30^ Wang's group demonstrated remarkable control over emulsion perturbation to generate bent shapes in micromotors, enabling precise manipulation of their geometry.^31^ By adjusting the temperature during synthesis, they produced different silica rod bending angles, which directly influenced propulsion modes such as linear movement, steering, and spinning.

There has been no comparative study to investigate the locomotion performance of self-propelled MMs designed to have the same active surface areas but different shapes. Herein, we report the velocity and motion behavior of SU-8 MMs fabricated in different shapes (cylindrical, rectangular cuboid, triangular prism, pentagonal prism and pentagrammic prism) and deposited with zinc (Zn). We specifically focus on examining how different MM shapes affect their motion in an acid solution while earlier research from different groups has explored the fundamental aspects of Zn-based MMs in an HCl environment or broad performance of MMs.^32–36^ We provide a detailed comparison of the average velocities of differently shaped MMs with the same height and the same surface area covered by the catalyst. In this study, we aimed to isolate the effect of MM shape on velocity by keeping the surface area covered by the catalyst constant. Excluding this factor, we made sure that any variation in velocity could be directly linked to the shape of the MMs. The results of our study shed new light on the relation between the shape of MMs and self-diffusiophoresis efficiency.

We selected the most frequently used photosensitive polymer (SU-8) for microfabrication due to capability to produce MMs with high aspect ratios, strong adhesion properties and biocompatible nature which make it suitable for biomedical applications.^37^

As shown in Scheme 1, self-propelled microdevices were fabricated on silicon carrier substrates using a combination of maskless lithography in SU-8 and thermal evaporation of Zn. Prior to the SU-8 lithography, a thin anti-adhesion layer consisting of 5 nm Ti and 20 nm Au was deposited to facilitate the detachment of the final MMs from the substrate. Ti is widely recognized as a material with excellent adhesion characteristics, particularly in bonding the Au layer to the Si substrate, while Au forms an inert surface that allows for easy detachment of the micromotors from the substrate without damage.^7^ Their combination makes an excellent choice for the anti-adhesion layer based on adhesion properties, biocompatibility, and stability.

**Scheme 1:**
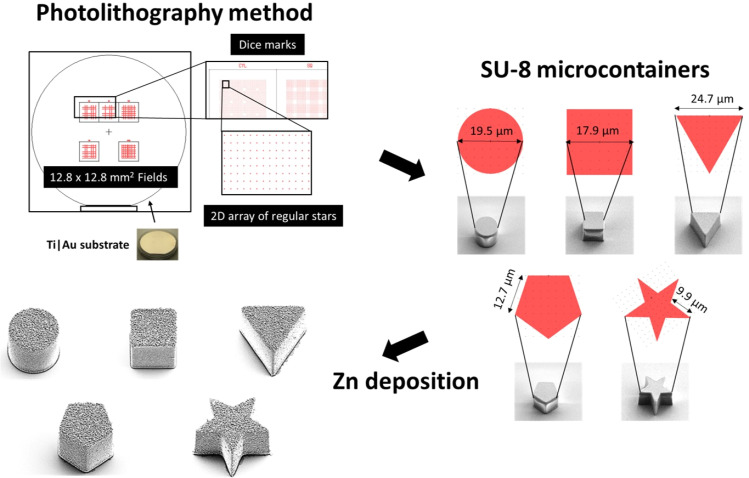
Schematic representation of the fabrication of Zn/SU-8 micromotors (MMs).

During the UV exposure, cylindrical, rectangular cuboid, triangular prism, pentagonal prism and pentagrammic prism were defined in approximately 19 μm thick SU-8. The overall aim was to produce MMs with different geometries all having the same top surface area.

The different micromotor geometries were exposed in separate 12.8 × 12.8 mm^2^ fields (Scheme 1). Each field contains 3600 MMs arranged in a regular 2D array, with a period of 150 μm in both directions.

A total of three substrates (Ti|Au release) were prepared with MMs defined in SU-8 with a thickness of approximately 19 μm. Five different fields representing five different shapes (cylindrical, rectangular cuboid, triangular prism, pentagonal prism, and pentagrammic prism) were prepared on each substrate, as shown in Fig. S1A.† All fields on all substrates were characterized by optical microscopy Fig. S1B,† Table S1† and vertical scanning interferometry (VSI) Table S2.† Based on the VSI data, it can be seen that the average height is slightly less than 19 μm, but the height is not subject to large variations. From the data collected by bright-field optical microscopy, it is clear that there is minimal variation in the measured lateral length/diameters of the different geometries.

The top surface of the fabricated MMs was deposited with Zn in a thermal evaporator. We investigated the surface structure and morphology of the Zn/SU-8 MMs using Scanning electron microscopy (SEM). From Fig. 1, five different MMs shapes were observed: cylindrical (Fig. 1A), rectangular cuboid (Fig. 1B), triangular prism (Fig. 1C), pentagonal prism (Fig. 1E) and pentagrammic prism (Fig. 1F). The Zn/SU-8 MMs made in all five shapes are carefully designed so that they all have the same top surface area covered by Zn (314 μm^2^) and a height of approximately ∼19 μm. The aim of this was to establish the same aspect ratio of length to average surface area for all studied MMs. Top and bottom views of the Zn/SU-8/Triangular prism MMs are displayed in Fig. 1D and H, respectively. These two images illustrate the surface roughness and distinct hexagonal grains or flakes on the top surface of the MMs corresponding to the Zn layer, and no Zn is present on the bottom view. They also confirm that Zn is successfully deposited on the SU-8 structures. Due to the rough and grainy appearance of the deposited Zn, the physical thickness of the layer is substantially larger than the 100 nm set point. The Zn film on the horizontal surface of the substrate has an irregular and non-planar appearance (Fig. 1G). Energy-dispersive X-ray spectroscopy (EDX) data indicate that there is 77 wt% Zn on the top surface of the Zn/SU-8 MMs.

**Fig. 1:**
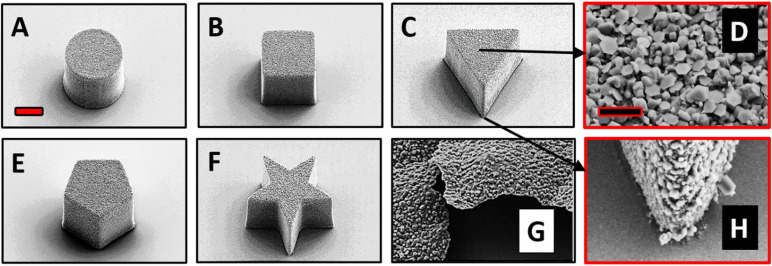
Characterization of Zn/SU-8 MMs. SEM image of (A) cylindrical, (B) rectangular cuboid, (C) triangular prism, (D) top view of triangular prism, (E) pentagonal prism, (F) pentagrammic prism, (G) Zn film and (H) bottom view of triangular prism MMs.

We focused our research on identifying differences in the velocities of the Zn/SU-8 MMs. In this regard, we turned our attention to the performance of these Zn/SU-8 MMs in an acidic solution. It is well known that Zn-based MMs exhibit efficient movement in acidic solutions because they strongly react with acid, generating hydrogen bubbles.^38^ To reveal the movement of the Zn/SU-8 MMs in an acidic solution, their velocity was monitored in a strongly acidic medium (1 M HCl, 1.3% Triton X-100). HCl was selected as a fuel based on its widespread use and effectiveness in generating propulsion for MMs in similar systems.^39,40^ Specifically, prior work demonstrated that 1.8 M HCl is an efficient fuel for propelling Zn-Janus MMS.^41^ In our experiments, we tested various concentrations and found that while 1.8 M HCl generates robust propulsion, 1 M HCl is the minimum concentration required to initiate consistent micromotor movement. This observation made 1 M HCl the optimal concentration choice for our study.

First, it was observed that non-coated SU-8 microcontainers showed only random Brownian motion when dispersed in 1 M HCl. However, when they were coated with 100 nm Zn on top, autonomous motion was observed (Fig. 2). To understand the MMs propulsion behavior, both bubble propulsion and the self-diffusiophoresis mechanism could be used as an explanation. As mentioned earlier, Zn-based MMs in contact with the HCl environment generate bubbles that are visible in Fig. 3B. However, this bubble-propelled mechanism is not responsible for the motion of the MMs. The asymmetric structure of the Zn/SU-8 MMs results in the asymmetric release of hydrogen and the formation of zinc chloride which creates a concentration gradient around the MMs. The MMs propel themselves primarily *via* the self-diffusiophoretic mechanism. The produced Zn/SU-8 MMs (100 nm Zn layer) displayed different velocities from 11.54 to 18.75 μm s^−1^ at the same fuel concentration (Fig. 2, green bars). Zn/SU-8/Rectangular cuboid MMs exhibited a 62% faster velocity than Zn/SU-8/Cylindrical MMs (average velocity 11.54 μm s^−1^), and a 43% faster movement compared to Zn/SU-8/Pentagrammic prism MMs (average velocity 12.54 μm s^−1^). These results are confirmed by statistical analysis; the differences between the Zn/SU-8/Rectangular cuboid MMs and the Zn/SU-8/Cylindrical MMs and the Zn/SU-8/Rectangular cuboid and Zn/SU-8/Pentagrammic prism MMs are both statistically significant (*p*-value = 1.51 × 10^−7^ and *p*-value = 7.35 × 10^−7^, respectively). Zn/SU-8/Pentagonal prism MMs and Zn/SU-8/Triangular prism MMs move at average velocities of 13.31 and 16.45 μm s^−1^, respectively. The experimental data align closely with the theoretical predictions. Considering that three main factors—symmetry,^42,43^ geometry,^44,45^ and hydrodynamic drag force^46^—affect the velocity of the MMs, it was expected that the Zn/SU-8/Rectangular cuboid MMs would move faster than other studied MMs, despite having the same active surface area covered with Zn.^47,48^ The Zn/SU-8/Rectangular cuboid MMs benefit from simple geometry and planar symmetry, resulting in more uniform and predictable drag characteristics. In contrast, Zn/SU-8/Pentagrammic prism MMs have a complex design with five non-parallel faces and angular corners, leading to more chaotic and disturbed fluid movement. Although the Zn/SU-8/Pentagonal prism MMs have a symmetric base, their five vertices and edges cause irregular flow patterns. The asymmetry of the triangular base in Zn/SU-8/Triangular prism MMs results in elevated drag forces.^49^ Similarly, the Zn/SU-8/Pentagonal prism MMs exhibit higher velocity than the Zn/SU-8/Pentagrammic prism MMs due to their less complex and more streamlined design. Their simpler geometry could result in reduced drag force and less turbulence.^50,51^ Rectangular cuboid MMs and triangular prism MMs have fewer surfaces and sharper edges than pentagonal prism MMs. Pentagonal prism MMs have a more complex structure due to their five-sided base, with internal angles of 108°, whereas the rectangular cuboid MMs have angles of 90° and the triangular prism MMs have angles of 60°. The pronounced edges in rectangular cuboid MMs and triangular prism MMs enhance localized disturbances in the surrounding fluid, improving self-diffusiophoresis and facilitating more streamlined fluid interactions. Compared to pentagrammic prism MMs, rectangular cuboid MMs and triangular prism MMs have fewer surfaces and less complex geometry. Although pentagrammic prism MMs have sharper edges, their complex geometry (irregular shape and asymmetric angles of the pentagrammic base) causes them to move slower than rectangular cuboid and triangular prism MMs. Additionally, the larger surface area and sharper edges of the pentagrammic prism amplify fluid resistance, further reducing its velocity.

**Fig. 2:**
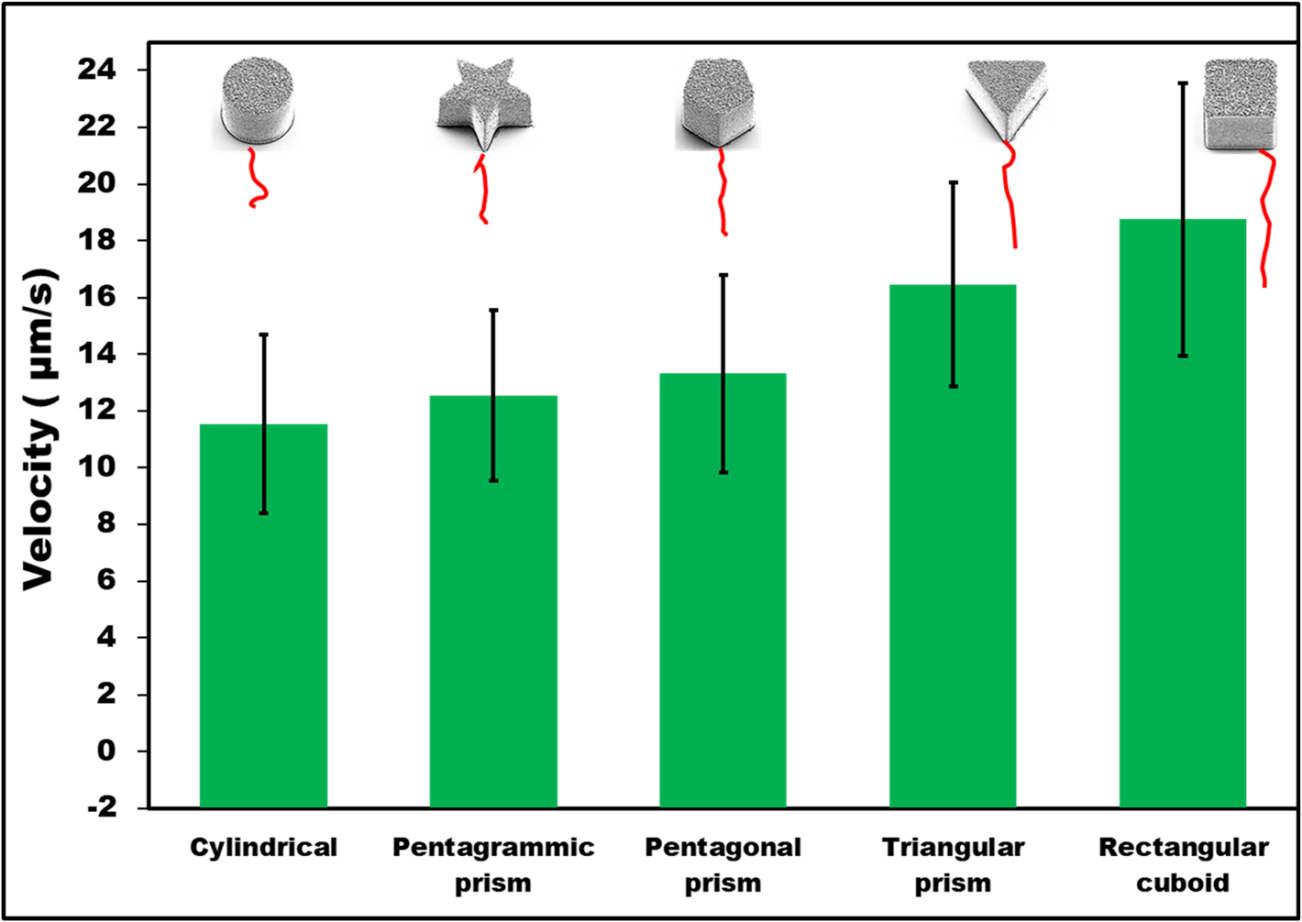
Average velocities of Zn/SU-8 MMs with 100 nm Zn thickness (error bars represent standard deviations from 25 independent measurements. Red lines are representative examples of trajectories).

**Fig. 3:**
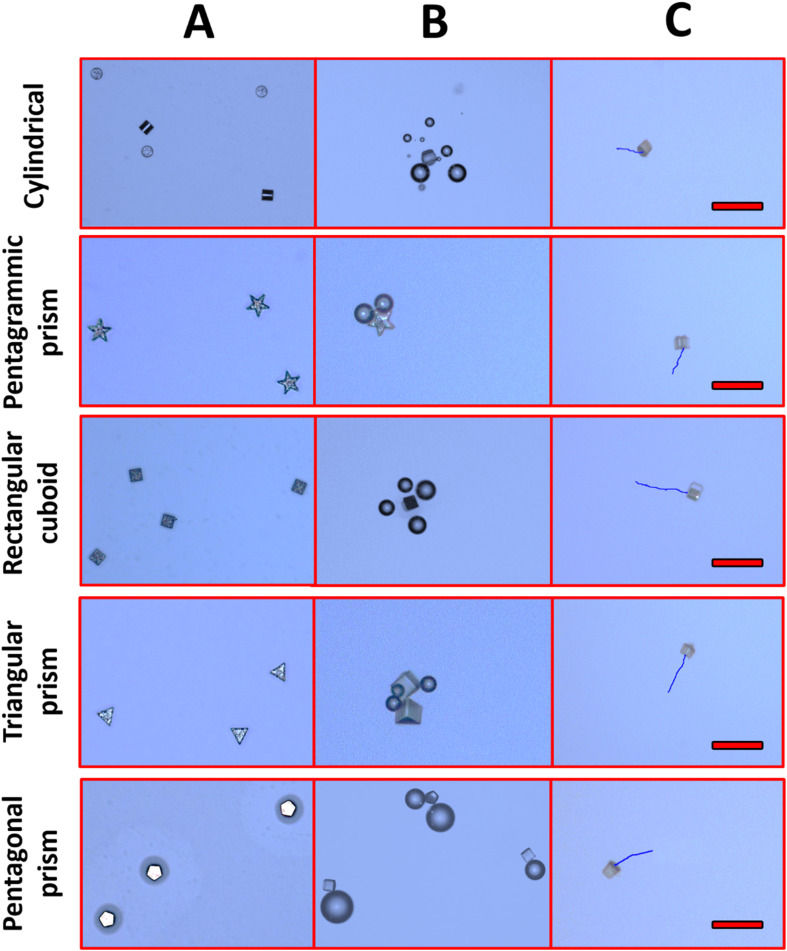
Optical imaging of Zn/SU-8 MMs (A) before acid addition, (B) bubble formation after acid addition (1 M HCl, 1.3% Triton X-100), (C) tracking over time duration of 5 s. Scale bars: 100 μm.

Compared to Zn/SU-8/Rectangular cuboid, Zn/SU-8/Triangular prism, Zn/SU-8/Pentagonal prism, and Zn/SU-8/Pentagrammic prism MMs, Zn/SU-8/Cylindrical MMs exhibit slower velocity due to their smooth, uniform surface, which creates less favorable fluid dynamics for MMs propulsion. Specifically, due to their continuous surface promotes laminar flow around their surfaces and increases drag forces that hinder propulsion.^52^ The geometry of Zn/SU-8/Cylindrical MMs features a lower perimeter-area ratio compared to the other studied shapes, reducing the surface area available for interactions with the acid medium, which is crucial for effective ion release. As a result, the self-diffusiophoresis mechanism is less effective in cylindrical designs compared to more intricate geometries that generate better propulsion.

Bishop and coworkers conducted a study on shape-controlled MMs, focusing on Pt-based MMs with twisted star shapes, which were fabricated using projection lithography.^24^ In contrast to our research, their study explored how the type and degree of shape asymmetry influence the prediction of both the direction (clockwise or counterclockwise) and the rotational speed of MMs. They also emphasized the need for more selective catalysts that can operate more efficiently at lower concentrations of hydrogen peroxide to enhance the overall performance of the motors.

Apart from velocity calculation, we also evaluated the MMs using optical microscopy (Fig. 3A). We observed no bubble formation in the absence of fuel. In contrast, after fueling (1 M HCl, 1.3% Triton X-100), MMs of all shapes produced bubbles (Fig. 3B). The Zn compartment of MMs reacts with fuel, generating bubbles. Even though the bubble formation plays a role in initiating the MMs movement, our findings show that the self-diffusiophoresis mechanism is primarily responsible for their driving. Zn layer (100 nm) is consumed relatively quickly (within 2–3 seconds), causing the MMs to stop generating bubbles. This transition is shown in Fig. 3C, where the linear motion of the MMs is visible after the bubbles have dissipated, indicating that self-diffusiophoresis drives the movement beyond the initial bubble propulsion phase. This indicates that self-diffusiophoresis is the key mechanism for the motion of MMs. Moreover, the ZnCl_2_ gradients lead to self-diffusiophoresis due to the nature of the ion release from the Zn surface during its interaction with the acid. Specifically, when the Zn part of MMs reacts with 1 M HCl, Zn^2+^ ions are released, forming a local concentration gradient. This gradient creates local electrochemical potential differences, establishing diffusive gradients that drive MMs locomotion.^53,54^ The effectiveness of self-diffusiophoresis hinges on the continual formation of these gradients, generating fluid movement surrounding the MMs. This movement enables forward propulsion without the need for bubble propulsion. Fig. 3C represents the tracking performance of the MMs' movement.

In this work, we employed cylindrical, rectangular cuboid, triangular prism, pentagonal prism and pentagrammic prism-shaped Zn/SU-8 MMs to investigate their motion performance when exposed to an acidic environment. Their average velocities were studied and compared under the same conditions (1 M HCl, 1.3% Triton X-100). Zn/SU-8/Cylindrical, Zn/SU-8/Rectangular cuboid, Zn/SU-8/Triangular prism, Zn/SU-8/Pentagonal prism, and Zn/SU-8/Pentagrammic prism MMs generated bubbles in acidic solution but exhibited motion through a self-diffusiophoretic mechanism. Zn/SU-8/Rectangular cuboid MMs achieved the fastest average velocity ∼18.75 μm s^−1^, while Zn/SU-8/Cylindrical MMs displayed the slowest average velocity ∼1.54 μm s^−1^. It can be concluded that rectangular cuboid and triangular prism shaped MMs have better motion properties and thus higher velocities than cylindrical, pentagrammic prism and pentagonal prism-shaped MMs for the same top surface area covered by Zn and same length. Due to the relatively simple synthesis process and low cost, shape-optimized Zn/SU-8 MMs hold significant potential to enhance efficiency and navigation in both environmental and medical applications by enabling precise control over movement. These findings could enable more effective MMs navigation in biological environments, improving the accuracy of drug delivery to specific areas of the body. In environmental applications, shape-optimized MMs could traverse faster through polluted water, speeding up the removal of harmful components. Future efforts will involve the fabrication of MMs with strategically designed shapes and drug-loading cavity at the base, followed by testing in complex biological media under different pH and temperature conditions.

## Experimental part

### Micromotor fabrication

The MMs were produced by maskless lithography in the epoxy-based negative photoresist SU-8 (SU-8 2015, micro resist technology GmbH, Berlin Germany) followed by thermal evaporation of Zn. Single side polished silicon wafers (Topsil Globalwafers A/S, Frederikssund, Denmark) having a diameter of 100 mm and a thickness of 525 μm were used as carrier substrates. Prior to the lithography process, an anti-adhesion layer composed of 5 nm Ti and 20 nm Au was deposited on the silicon substrates by electron-beam evaporation (Temescal FC-2000, Ferrotec Corporation, Santa Clara, CA, USA). The anti-adhesion layer allows easy detachment of the produced SU-8 MMs after the lithography and Zn evaporation steps. Hereafter, an approximately 20 μm thick SU-8 layer was spin coated (RCD8 semi-automated spin coater, Süss Microtec, Garching, Germany) on the carrier substrates which were subsequently soft baked on a hotplate at 50 °C for 1.5 h. The thin SU-8 layer was then selectively exposed using a maskless aligner (MLA100 Tabletop Maskless Aligner, Heidelberg Instruments, Heidelberg, Germany) with an illumination wavelength of 365 nm. The five discrete fields, having MMs with different geometries, where exposed using a dose of 175 mJ cm^−2^ and cross-linking was promoted in a post exposure bake at 50 °C for 6 h whereafter the structures were developed in mr-Dev 600 (micro resist technology GmbH, Berlin Germany) by immersion in two dedicated baths for 4 min. Finally, the developed MMs were subject to a gentle isopropanol flush and left to dry. The topography inspection was done using a combination of bright-field microscopy (Nikon Eclipse L200, Nikon Metrology, Tokyo, Japan) and vertical scanning interferometry (PLu Neox 3D Optical Profiler, Sensofar Metrology, Barcelona, Spain) for interrogating the horizontal and vertical dimensions of the produced MMs. As the adhesion between thermally evaporated zinc and SU-8 is relatively poor, an interface metal stack composed of 5 nm Ti and 20 nm Au was deposited on the MMs by electron-beam evaporation. The final Zn deposition was done using a thermal evaporator (Kurt J. Lesker Nano 36, Kurt J. Lesker Company, Jefferson Hills, PA, USA) equipped with an Al_2_O_3_ crucible and a quartz crystal microbalance for monitoring the deposition thickness. The deposition process was done using a low rate of 0.5 Å s^−1^ and an initial chamber pressure of 1–3 × 10^−6^ Torr. Refer to Table S3† for a detailed outline of the MMs fabrication process. After thermal evaporation, the samples were inspected using conventional scanning electron microscopy (SEM, Zeiss Supra 60 VP, Carl Zeiss AG, Oberkochen, Germany) and energy-dispersive X-ray analysis (EDX). Prior to the final SEM/EDX inspection, the bulk Zn layer residing on the horizontal surfaces of the silicon carrier substrate was removed using a nitrogen gun. The “dry lift-off” was easily achieved since the adhesion between the first Ti|Au stack (used to ensure detachment of the SU-8 MMs) and the Ti|Au|Zn is intrinsically poor. This allowed for removing the bulk metal film from the entire surface area not covered by SU-8 MMs. After the final inspection, the substrates were scribed into five discrete chips, each containing 3600 MMs with different geometries.

### Motion study

To study the relationship between the velocity of prepared MMs and their shape, all MMs were propelled in an acid solution (1 M HCl, 1.3% Triton X-100). A Leica INM100 optical microscope (Leica, Germany) was used to capture the videos, and NIS Elements Software D version 4.20 (Nikon, Japan) was used to analyze the data. Average velocities were measured based on 20 recorded videos. To determine the statistical difference of the collected data, a two-tailed *t*-test was conducted.

## Data availability

The data supporting this article have been included as part of the ESI.†

## Conflicts of interest

There are no conflicts to declare.

## Supplementary Material

NA-OLF-D4NA00721B-s001

NA-OLF-D4NA00721B-s002

## References

[cit1] Lyu X., Liu X., Zhou C., Duan S., Xu P., Dai J., Chen X., Peng Y., Cui D., Tang J., Ma X., Wang W. (2021). J. Am. Chem. Soc..

[cit2] Noh W., Jo S., Kim J., Lee T. S. (2021). Langmuir.

[cit3] Cao W., Liu Y., Ran P., He J., Xie S., Weng J., Li X. (2021). ACS Appl. Mater. Interfaces.

[cit4] Xu H., Medina-Sánchez M., Schmidt O. G. (2020). Angew. Chem., Int. Ed..

[cit5] Guo J., Kim K., Lei K. W., Fan D. L. (2015). Nanoscale.

[cit6] Orozco J., Jurado-Sánchez B., Wagner G., Gao W., Vazquez-Duhalt R., Sattayasamitsathit S., Galarnyk M., Cortés A., Saintillan D., Wang J. (2014). Langmuir.

[cit7] Maric T., Adamakis V., Zhang Z., Milián-Guimerá C., Thamdrup L. H. E., Stamate E., Ghavami M., Boisen A. (2023). Small.

[cit8] Cao W., Wei W., Qiu B., Liu Y., Xie S., Fang Q., Li X. (2024). Chem. Eng. J..

[cit9] Mena-Giraldo P., Kaur M., Maurizio S. L., Mandl G. A., Capobianco J. A. (2024). ACS Appl. Mater. Interfaces.

[cit10] Gao Y., Ou L., Liu K., Guo Y., Li W., Xiong Z., Wu C., Wang J., Tang J., Li D. (2024). Angew. Chem., Int. Ed..

[cit11] Xu X., Huo Z., Guo J., Liu H., Qi X., Wu Z. (2020). Bio-Des. Manuf..

[cit12] Mayorga-Martinez C. C., Zhang L., Pumera M. (2024). Chem. Soc. Rev..

[cit13] Jurado-Sánchez B., Wang J. (2018). Environ. Sci.:Nano.

[cit14] Jing D., Li Z., Yan W., Zhang J., Guo Y. (2024). New J. Chem..

[cit15] Mei Y., Solovev A. A., Sanchez S., Schmidt O. G. (2011). Chem. Soc. Rev..

[cit16] Zhao G., Viehrig M., Pumera M. (2013). Lab Chip.

[cit17] Huang W., Manjare M., Zhao Y. (2013). J. Phys. Chem. C.

[cit18] Liu L., Bai T., Chi Q., Wang Z., Xu S., Liu Q., Wang Q. (2017). Micromachines.

[cit19] Wang D., Guan D., Su J., Zheng X., Hu G. (2021). Phys. Fluids.

[cit20] Kang E., Lee W., Lee H. (2023). J. Phys. Chem. Lett..

[cit21] Wang L., Chen J., Feng X., Zeng W., Liu R., Lin X., Ma Y., Wang L. (2016). RSC Adv..

[cit22] Maric T., Moo J. G. S., Khezri B., Sofer Z., Pumera M. (2017). Appl. Mater. Today.

[cit23] Sanchez S., Ananth A. N., Fomin V. M., Viehrig M., Schmidt O. G. (2011). J. Am. Chem. Soc..

[cit24] Brooks A. M., Tasinkevych M., Sabrina S., Velegol D., Sen A., Bishop K. J. M. (2019). Nat. Commun..

[cit25] Wang H., Moo J. G. S., Pumera M. (2016). ACS Nano.

[cit26] Su M., Liu M., Liu L., Sun Y., Li M., Wang D., Zhang H., Dong B. (2015). Langmuir.

[cit27] Zhang L., Xiao Z., Chen X., Chen J., Wang W. (2019). ACS Nano.

[cit28] Zhang Q., Dong R., Wu Y., Gao W., He Z., Ren B. (2017). ACS Appl. Mater. Interfaces.

[cit29] Wang Z., Shang L., Gao Z., Chan K. K., Gong C., Wang C., Xu T., Liu T., Feng S., Chen Y. C. (2022). Lab Chip.

[cit30] Maric T., Løvind A., Zhang Z., Geng J., Boisen A. (2023). Adv. Healthcare Mater..

[cit31] Mu Y., Duan W., Hsu K. Y., Wang Z., Xu W., Wang Y. (2022). ACS Appl. Mater. Interfaces.

[cit32] Ahmed S., Wang W., Bai L., Gentekos D. T., Hoyos M., Mallouk T. E. (2016). ACS Nano.

[cit33] Sabrina S., Tasinkevych M., Ahmed S., Brooks A. M., Olvera De La Cruz M., Mallouk T. E., Bishop K. J. M. (2018). ACS Nano.

[cit34] Maggi C., Saglimbeni F., Dipalo M., De Angelis F., Di Leonardo R. (2015). Nat. Commun..

[cit35] Magdanz V., Stoychev G., Ionov L., Sanchez S., Schmidt O. G. (2014). Angew. Chem., Int. Ed..

[cit36] Gibbs J. G., Kothari S., Saintillan D., Zhao Y. P. (2011). Nano Lett..

[cit37] Lorenz H., Despont M., Vettiger P., Renaud P. (1998). Microsyst. Technol..

[cit38] Esteban-Fernández de Ávila B., Angsantikul P., Li J., Gao W., Zhang L., Wang J. (2018). Adv. Funct. Mater..

[cit39] Gao W., D'Agostino M., Garcia-Gradilla V., Orozco J., Wang J. (2013). Small.

[cit40] Zhang L., Zhang H., Liu M., Dong B. (2016). ACS Appl. Mater. Interfaces.

[cit41] Chen C., Karshalev E., Li J., Soto F., Castillo R., Campos I., Mou F., Guan J., Wang J. (2016). ACS Nano.

[cit42] Yu T., Chuphal P., Thakur S., Reigh S. Y., Singh D. P., Fischer P. (2018). Chem. Commun..

[cit43] Xiong K., Lin J., Chen Q., Gao T., Xu L., Guan J. (2023). Matter.

[cit44] Wang H., Chen X., Meng X., He Y., Jin B., Zhao X., Ye C. (2024). Chem. Mater..

[cit45] Zhang C., Wang Y., Chen Y., Ma X., Chen W. (2022). Nanomaterials.

[cit46] Liu T., Xie L., Price C. A. H., Liu J., He Q., Kong B. (2022). Chem. Soc. Rev..

[cit47] Li L., Wang J., Li T., Song W., Zhang G. (2014). Soft Matter.

[cit48] Ning H., Zhang Y., Zhu H., Ingham A., Huang G., Mei Y., Solovev A. A. (2018). Micromachines.

[cit49] Mohammadi B., Pironneau O. (2004). Annu. Rev. Fluid. Mech..

[cit50] Yang Q., Xu L., Zhong W., Yan Q., Gao Y., Hong W., She Y., Yang G. (2020). Adv. Intell. Syst..

[cit51] Li L., Wang J., Li T., Song W., Zhang G. (2014). Soft Matter.

[cit52] Frankel A. E., Khair A. S. (2014). Phys. Rev. E:Stat., Nonlinear, Soft Matter Phys..

[cit53] Kichatov B., Korshunov A., Sudakov V., Golubkov A., Smovzh D., Sakhapov S., Skirda M. (2024). Phys. Chem. Chem. Phys..

[cit54] Zhou C., Zhang H. P., Tang J., Wang W. (2018). Langmuir.

