# Supplementary material for: Shape-controlled movement of Zn/SU-8 micromotors
Source: Nanoscale Adv. 2024 Nov 25;6(24):6134–41. doi: 10.1039/d4na00721b (PMC11587533; doi:10.1039/d4na00721b)
Supplement: NA-006-D4NA00721B-s002 [file NA-006-D4NA00721B-s002.pdf]

Supplementary Information (SI) for Nanoscale Advances.
This journal is © The Royal Society of Chemistry 2024

Supporting Information

Shape-controlled movement of Zn/SU-8 micromotors

Tijana Maric[ab]*, Lasse Højlund Eklund Thamdrup[ab], Anja Boisen[ab]

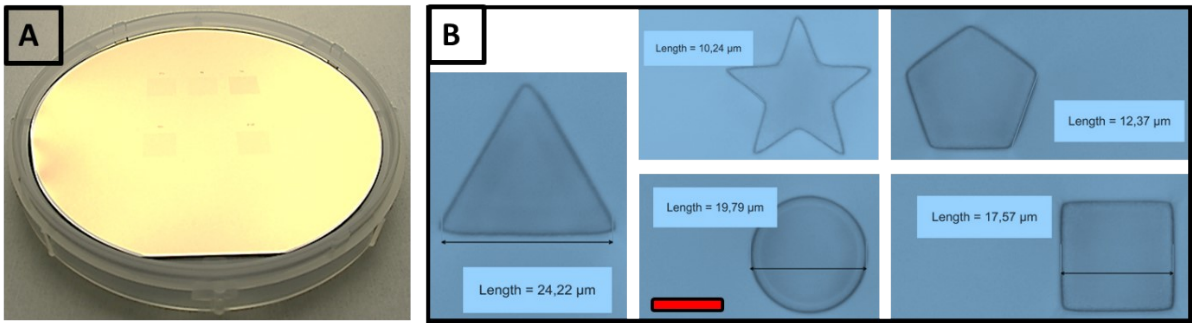

Figure S1. A) One substrate with 5 fields contain cylindrical, square, triangular, pentagon and star shape MMs. B) Optical microscopy images of all 5 fields. Scale bar: 10 µm.

Table S1. Summarized data for the diameter or side length of Zn/Su-8 MMs calculated from the data collected by optical microscope.

	Diameter or side length [µm]				
	**Cylindrical**	**Rectangular cuboid**	**Triangular prism**	**Pentagonal prism**	**Pentagrammic prism**
Sharp 1 Ti|Au	19.5	18.0	25.0	9.7	12.5
Sharp 2 Ti|Au	19.5	17.9	24.7	9.9	12.7
Sharp 3 Ti|Au	19.4	17.6	24.2	10.1	13.0
**AVERAGE**	**19.5**	**17.8**	**24.6**	**9.9**	**12.7**

Table S2. Summarized vertical scanning interferometry (VSI) data for the height of the Zn/Su-8 MMs.

	Height[μm]				
	**Cylindrical**	**Rectangular cuboid**	**Triangular prism**	**Pentagonal prism**	**Pentagrammic prism**
Sharp 1 Ti|Au	18.5	18.2	18.6	19.0	19.0
Sharp 2 Ti|Au	18.1	18.3	18.3	18.8	18.7
Sharp 3 Ti|Au	18.3	18.0	18.2	18.8	18.7
**AVERAGE**	**18.3**	**18.2**	**18.4**	**18.9**	**18.8**

Table S3. Preparation steps for Zn/Su-8 MMs fabrication.

Description	Equipment	Parameters
WAFER PREPARATION		
Anti-Adhesion layer	Temescal	Ti 5 nm (5 Å/s), Au 20 nm (10 Å/s)
SU-8 FIRST LAYER SPINNING		
SU-8 spin-coating	Spin Coater: LabSpin 03	500rpm, 15s, 200rpm/s
SU-8 soft-bake	Hotplate1	1.5h, 50ºC
SU-8 FIRST LAYER EXPOSURE		
SU-8 exposure	Aligner: Maskless 01	D=175 mJ/cm^2
Post-exposure bake	Hotplate1	6h, 50ºC
OM inspection	Nikon ECLIPSE L200	
SU-8 DEVELOPMENT		
SU-8 development	mr-Dev 600	4min
Rinse	Developer SU-8	Isopropanol, Air
Characterization	Nikon ECLIPSE L200<br>PLu Neox 3D Optical Profiler	
Dicing	Dicing saw	Pitch 12.8 mm, 10 mm/s
Interface metal stack	Electron-beam evaporator	Ti 5 nm, Au 20 nm
DEPOSITION		
Zn deposition	Thermal evaporator equipped with an Al_2O_3 crucible and a quartz crystal microbalance	0.5 Å/s, 1-3×10^{-6} Torr
